# New Solvent and Coagulating Agent for Development of Chitosan Fibers by Wet Spinning

**DOI:** 10.3390/polym13132121

**Published:** 2021-06-28

**Authors:** Ghasem Mohammadkhani, Sunil Kumar Ramamoorthy, Karin H. Adolfsson, Amir Mahboubi, Minna Hakkarainen, Akram Zamani

**Affiliations:** 1 Swedish Centre for Resource Recovery, University of Borås, 501 90 Borås, Sweden; gh.mkhani@gmail.com (G.M.); sunil_kumar.lindstrom_ramamoorthy@hb.se (S.K.R.); amir.mahboubi_soufiani@hb.se (A.M.); 2 Department of Fibre and Polymer Technology, KTH Royal Institute of Technology, 100 44 Stockholm, Sweden; karinad@kht.se (K.H.A.); minna@kth.se (M.H.)

**Keywords:** monofilament, chitosan, adipic acid, wet spinning, sulfuric acid, coagulation bath

## Abstract

Adipic acid was evaluated as a novel solvent for wet spinning of chitosan fibers. A solvent with two carboxyl groups could act as a physical crosslinker between the chitosan chains, resulting in improved properties of the fibers. The performance of adipic acid was compared with conventional solvents, i.e., lactic, citric, and acetic acids. Chitosan solutions were injected into a coagulation bath to form monofilaments. Sodium hydroxide (NaOH) and its mixture with ethanol (EtOH) were used as coagulation agents. Scanning electron microscopy confirmed the formation of uniform chitosan monofilaments with an even surface when using adipic acid as solvent. These monofilaments generally showed higher mechanical strength compared to that of monofilaments produced using conventional solvents. The highest Young’s modulus, 4.45 GPa, was recorded for adipic acid monofilaments coagulated in NaOH-EtOH. This monofilament also had a high tensile strength of 147.9 MPa. Furthermore, taking advantage of chitosan insolubility in sulfuric acid (H_2_SO_4_) at room temperature, chitosan fibers were successfully formed upon coagulation in H_2_SO_4_-EtOH. The dewatering of fibers using EtOH before drying resulted in a larger fiber diameter and lower mechanical strength. Adipic acid fibers made without dehydration illustrated 18% (for NaOH), 46% (for NaOH-EtOH), and 91% (for H_2_SO_4_-EtOH) higher tensile strength compared to those made with dehydration.

## 1. Introduction

Chitin, the second most abundant polysaccharide in nature, can be found naturally as ordered crystalline microfibrils forming structural components in the exoskeleton of arthropods and the fungal cell wall. Chitosan, the most famous derivative of chitin, is a copolymer of glucosamine (GlcN) and N-acetyl-glucosamine (GlcNAc) units where GlcN is predominant [1]. Chitosan is not soluble in water due to its hydrogen bonds and hydrophobic interactions between the chain segments. However, it can be dissolved easily in dilute acidic solutions such as aqueous solutions of acetic acid, citric acid, lactic acid, malic acid, and formic acid, due to the presence of non-bonding pairs of electrons in the amino groups, which become protonated in acidic solutions. The solubility of chitosan in environmentally friendly and inexpensive solvents is a great advantage to produce chitosan-based fibers [2,3].

Chitosan shows a broad range of antimicrobial activity against Gram-positive and Gram-negative bacteria and fungi [4]. Moreover, chitosan fibers are biocompatible, biodegradable, and non-toxic. Promising features such as wound-healing acceleration and anti-inflammatory properties make chitosan an excellent material for variety of medical applications such as controlled drug delivery, covering material for the wound-healing acceleration, and surgical sutures [1,5,6]. Additionally, well-defined chitosan macrofibres, which were processed into textile structures (3D nonwoven structures, stable knitted, and woven textile fabrics) have been evaluated as textile scaffolds in regenerative medicine such as cartilage and bone tissue engineering [5], and rotary wet spun chitosan fibers were used to obtain torsional artificial muscle [6]. Moreover, chitosan can be degraded in vivo by several enzymes, mostly by lysozyme. Based on this property, absorbable surgical suture was produced using the wet spinning system, from chitosan incorporated with N-acetyl-D-Glucosamine (GlcNAc) [7]. Chitosan has also been used in the textile industry to produce manmade fibers and as textile fiber finishes, coatings, and textile auxiliaries [8]. In addition, chitosan has been used as a preservative, a packaging additive, a dietary supplement, and as a nutrient encapsulation system in the food industry. Furthermore, in the agricultural industry, chitosan is used to protect plants from bacteria, fungi, and viruses, and as a fertilizer additive. Additionally, in the wastewater treatment industry, chitosan has been used to remove fats, dyes, and heavy metals, mostly due to its fat-binding and chelating properties [9]. The most common technique used to produce chitosan fibers is wet spinning. Chitosan spinnability is affected by several factors. Considering chitosan wet spinning, these factors include: the shear flow properties of the solution, the rate of coagulation in the bath, the extensional flow properties of the chitosan solution jet, and hydraulic drag of the threadline via the coagulation bath [10]. In wet spinning, the desired amount of chitosan is dissolved in a suitable solvent and directly extruded into a coagulation bath containing a liquid, which is miscible with the spinning solvent but a non-solvent for chitosan. This results in precipitated solid fiber in the solution [11,12].

A large number of coagulation systems like 1M sodium hydroxide (NaOH) [13], a mixture of 10% solutions of NaOH and ethanol (EtOH) [14], NaOH–sodium sulfate [15], NaOH–methanol, and calcium chloride or calcium acetate saturated water-methanol [16] have been evaluated in combination with different solvents such as acetic acid [12,17], lactic acid [18], formic acid [19], and glycine chloride [3] to prepare wet spun chitosan fibers. The crosslinking of chitosan fibers is usually performed to improve the mechanical properties of the fibers [20]. Most of the conventional acids used for the wet spinning of chitosan cannot directly act as a crosslinking agent. Therefore, post treatments using crosslinking agents are necessary to crosslink the chitosan fibers. In contrast, using di-functional carboxylic acids for the dissolution of chitosan brings the opportunity for ionic crosslinking of chitosan chains [21].

Chitosan is also soluble in adipic acid, an abundant, non-toxic, and biocompatible compound, and also the most important industrial dicarboxylic acid. Commercial adipic acid is mostly obtained from benzene [22] and is extensively used for the production of nylon 66 and polyurethane [21]. Recently, Niu et al. [23] reported the first example of direct biosynthesis of adipic acid from the lignin-derived compound. Moreover, the possibility of making chitosan hydrogels for, e.g., food texturing agents, nutraceutics, and controlled drug delivery, by utilizing different acids, including adipic acid, has been studied [24]. On the other hand, chitosan cannot be dissolved in dilute sulfuric acid (H_2_SO_4_) solutions at room temperature, but becomes soluble in hot boiling solutions [25].

The principal objective of the present work was to improve the process of wet spinning chitosan fibers to enhance the final fiber characteristics and properties. This was approached by the introduction of a novel solvent and a coagulant. Chitosan-adipic acid films have previously shown promising properties benefitting from the physical crosslinking of chitosan chains with adipic acid [21]. Therefore, we hypothesized that similar effects could be achieved by utilizing adipic acid as a solvent for the wet spinning of chitosan fibers. The production of chitosan monofilaments with adipic acid as a solvent was compared with three conventional solvents, i.e., lactic, citric and acetic acids. Past reports show that the chitosan can form crosslinks with sulfate ions, setting the stage for losing the solubility of chitosan in typical solvents such as acetic and lactic acids [26]. Thus, we anticipated that such a crosslinking could facilitate the chitosan coagulation and fiber formation. The performance of sulfuric acid as a new coagulation agent was compared to conventional coagulation agents, which were 1M NaOH and a 1:1 mixture of 10% solution of NaOH and EtOH. The influence of dewatering the monofilaments using ethanol was examined.

## 2. Materials and Methods

### 2.1. Materials

Chitosan (commercial medium Mw, DD 85%, viscosity 120 mPaS, and ash content less than 1%) was purchased from BioLog (Bremen, Germany), and used without further purification. The solvents acetic acid and citric acid anhydrous were obtained from Scharlau (Barcelona, Spain), while lactic acid (purity = 90%, water content = 10%) and adipic acid were purchased from Sigma-Aldrich (Tokyo, Japan and, Burlington, VT, USA, respectively). Sulfuric acid and sodium hydroxide pellets were acquired from Sigma-Aldrich (Stockholm, Sweden) and used for coagulation bath preparation. Ethanol absolute (VWR, Paris, France) was used for dewatering and for the preparation of the coagulation bath solutions.

### 2.2. Preparation of Spinning Solutions and Coagulation Baths

Chitosan solutions (4% *w*/*w*) were prepared by dissolving the chitosan powder in an aqueous solution of lactic acid and acetic acid (providing a stoichiometric amount of acids, 0.239 mol/L, for protonation of amino groups of chitosan). As adipic acid is dicarboxylic acid, 0.1195 (0.239/2) mol/L of acid was enough to dissolve the polymer. However, for citric acid, which has three carboxyl groups, chitosan was not dissolved in 0.0796 (0.239/3) mol/L of this acid. Therefore, 0.239 mol/L citric acid was used to dissolve the chitosan. The spinning solutions were maintained under mechanical stirring (600 rpm, ambient temperature) for 2 h. The solutions were then centrifuged (10 min, 5000× *g*) to eliminate the trapped air bubbles and stored at room temperature. Three different coagulation baths were prepared, including 1:1 mixture of 10% solution of NaOH and EtOH [14], 1 M NaOH [13], and 1:1 mixture of 2% solution of H_2_SO_4_, and EtOH.

### 2.3. Viscosity and pH Measurement

The measurements of solution viscosities and pH values were carried out at room temperature, using a sinewave vibro-viscometer (SV-10, A&D, Tokyo, Japan) and a pH meter (METTLER TOLEDO, Columbus, OH, USA), respectively.

### 2.4. Wet Spinning Procedure

**Figure 1:**
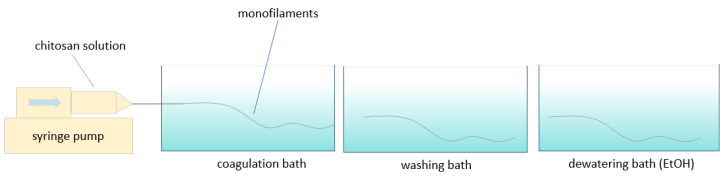
Schematic presentation of the wet spinning system.

The chitosan solutions were transferred to a 10 mL syringe and then injected into the coagulation bath using a syringe pump (WPI, Friedberg, Germany) at a fixed flow rate (1 mL/min) to form the chitosan monofilaments. The syringe needle (40 mm length, 0.8 mm diameter) was submerged directly into the bath via a hole (Figure 1). The residence time for coagulating was 2 min. Afterward, the filaments were transferred to the distilled water bath and ethanol bath in order to be washed and dewatered, respectively. To examine the effect of an ethanol bath on the properties of the monofilaments, samples prepared without the ethanol bath were examined as well. Finally, the monofilaments were collected from the last bath (water bath or ethanol bath) and dried at room temperature while fixed between two points on a whiteboard.

### 2.5. Characterization of the Chitosan Monofilaments

#### 2.5.1. Scanning Electron Microscopy (SEM)

The morphology of the chitosan fibers was analyzed using scanning electron microscopy. The images were acquired by high-resolution high-vacuum cold field emission Hitachi SEM S-4800 (Tokyo, Japan). Samples were coated with a 2 nm Pd/Pt layer and 3 kV acceleration voltage was used.

#### 2.5.2. Mechanical Properties

Tensile testing was used to characterize the mechanical properties of the chitosan monofilaments. Tensile tests were conducted on a Testing Machine (Tinius Olsen Ltd., Surrey, England) equipped with pneumatic grips and a load cell of 100 N under ambient conditions. The gauge length was 20 mm with a test speed of 20 mm/min. The average diameters of fibers were 0.165–0.293 mm, as determined by an axiosStar microscope Nikon SMZ800 (Kawasaki, Japan) and Samples were preconditioned for 24 h at 20 °C and 65% relative humidity prior to tensile testing [27]. Young’s modulus was obtained using the slope of stress–strain curves between 0.1 and 0.5% strain.

#### 2.5.3. Fourier Transform Infrared Spectroscopy (FTIR)

The molecular structures of commercial chitosan powder and wet spun chitosan monofilaments were examined by FTIR. The FTIR spectra were investigated with a Nicolet iS10 FTIR spectrometer (Thermo Scientific, Waltham, MA, USA) in transmittance mode over a wavenumber range of 600 to 4000 cm^−1^ at a resolution of 4 cm^−1^ through the accumulation of 32 scans for each spectrum. Samples were maintained in a desiccator for 3 h before the analysis.

#### 2.5.4. Differential Scanning Calorimetry (DSC)

DSC measurements were performed using DSC-Q2000 apparatus (TA Instruments, New Castle, DE, USA) to evaluate thermal behavior of fibers. Each sample (5–10 mg) was placed in a covered aluminum sample holder. An empty aluminum cell and lid was used as the reference for all measurements. The sample and reference pan were heated simultaneously from −30 °C to 230 °C under nitrogen atmosphere and heating rate of 10 °C/min. Nitrogen gas flow rate was fixed at 50 mL/min.

## 3. Results and Discussion

### 3.1. Fiber Formation

To evaluate the effect of different spinning solvents on the fiber properties, chitosan was dissolved in lactic acid (LA), acetic acid (AC), citric acid (CI), or adipic acid (AD). A stoichiometric amount of the acids for protonation of the amino groups of chitosan was applied. Viscosity and pH values of chitosan solutions in different acids are shown in Table 1. The chitosan aggregation is prevented when pH values are between 3.7 and 5.6 [5].

The different chitosan solutions were injected to the different coagulation baths and all monofilaments were washed with water after coagulation. All combinations are listed in Table 2. The monofilaments formed immediately, when the chitosan solutions entered the coagulation baths, and they could be collected manually. Interestingly, it was possible to form stable chitosan fibers with an even surface from the adipic acid solution. The preliminary experiments showed that using only H_2_SO_4_ as a coagulation bath resulted in the formation of unstable fibers which were impossible to collect from the bath, mainly due to breaking down during the fiber collection. However, a mixture of H_2_SO_4_ and EtOH as the coagulation bath significantly improved the fiber formation.

For all chitosan solutions, monofilaments coagulated in NaOH and NaOH-EtOH were collected easily. Conversely, it was harder to collect and fix the monofilaments coagulated in H_2_SO_4_-EtOH. The fibers formed in the bath containing H_2_SO_4_ absorbed more water than the other monofilaments and were less stable. For all chitosan solutions, monofilaments coagulated in NaOH and NaOH-EtOH could be stretched easily before drying. However, often the monofilaments formed by aggregation in H_2_SO_4_-EtOH were broken upon stretching. Among different acids tested, lactic acid resulted in fibers with higher stretchability in the wet form. Fibers obtained from adipic acid solutions showed proper spinnability and stretchability in the wet form, which means they were easily handled in coagulation bath and collecting process.

### 3.2. Morphology of Chitosan Monofilaments

**Figure 2:**
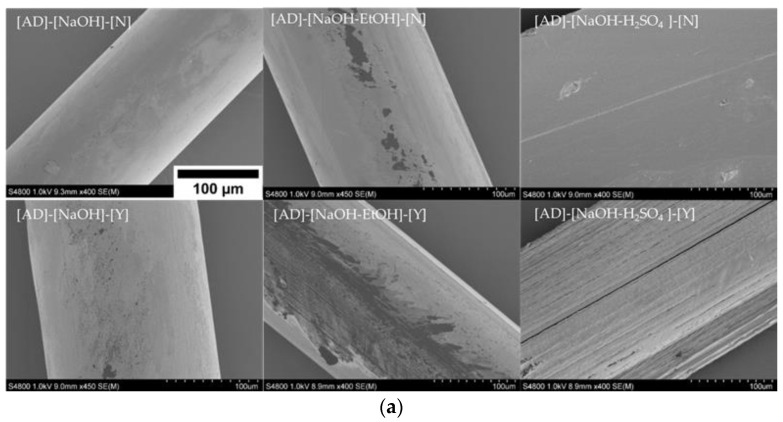

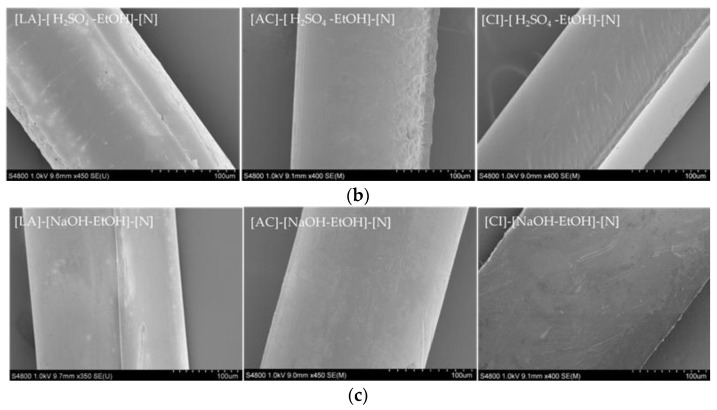
SEM images of chitosan monofilaments. Filaments prepared with (**a**) adipic acid as a solvent in different coagulation baths without and with dewatering; (**b**) lactic acid, acetic acid, or citric acid as solvents in H_2_SO_4_-EtOH bath without dewatering; and (**c**) lactic acid, acetic acid, or citric acid as solvents in NaOH-EtOH bath without dewatering. All images have been taken with the same magnification and a representative scale bar is shown in Figure 2a.

The SEM images of the wet spun monofilaments are presented in Figure 2. Fibers with round cross-sections were acquired in all cases. Figure 2a presents the fibers made using adipic acid as the solvent under different conditions (c.f. Table 2). Fibers made using NaOH as coagulation bath without dewatering showed a very uniform and smooth surface morphology, as well as smaller diameters compared to the other adipic acid monofilaments. In contrast, dewatering by EtOH damaged monofilaments’ surfaces to some extent, see for example the [AD]-[NaOH-EtOH]-[Y] sample. Knaul et al. [17] also reported a similar effect of the drying agent on the chitosan fiber surface. The presence of surface irregularities could be attributed to the rapid dehydration using ethanol, resulting in porous areas. The presence of EtOH in the coagulation bath also resulted in a similar effect on the fibers as can be seen for [AD]-[NaOH-EtOH]-[N]. However, smaller irregularities were observed, probably due to the lower concentration of EtOH. On the other hand, the fiber made in H_2_SO_4_-EtOH without dewatering exhibited a rather smooth surface. This can be related to the higher affinity of the fibers for water absorption in the presence of H_2_SO_4_. Upon contact with sulfuric acid, the protonated amino groups of chitosan can form an ionic complex of chitosan-sulfate, which, even though it is not soluble in aqueous solutions anymore [26], has a higher interaction with water compared to the non-protonated amino groups of chitosan that are aggregated in the presence of NaOH. Moreover, the non-protonated form of chitosan in NaOH bath leads to the formation of stronger interactions along the chitosan chains and contributes to obtaining fibers with smaller diameters as well as higher stability in the wet form compared to the fibers made in H_2_SO_4_-EtOH bath [28].

**Figure 3:**
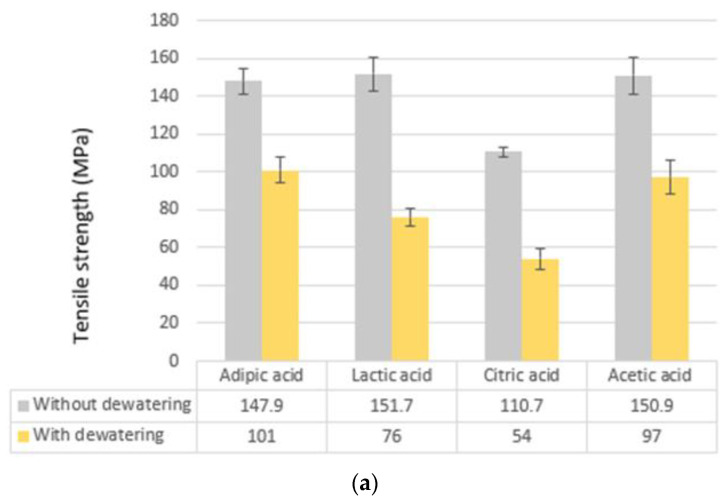

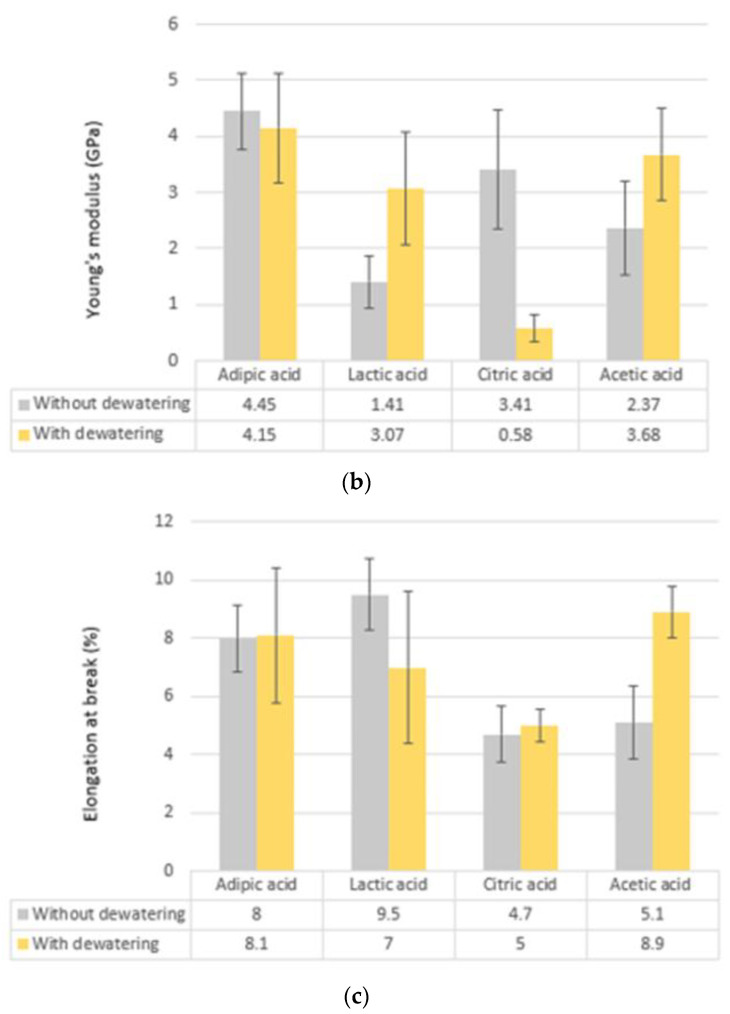
Mechanical properties of wet spun monofilaments coagulated in (1:1) 10% solution of NaOH and EtOH bath as the best coagulation bath: (**a**) tensile strength; (**b**) Young’s modulus; and (**c**) Elongation at break.

Figure 2b shows the monofilaments coagulated in H_2_SO_4_-EtOH bath using lactic acid, citric acid, and acetic acid as solvents, respectively. The images confirm that H_2_SO_4_ could be used as a part of an effective coagulation bath, contributing to fibers with a smooth and uniform surface. As reported earlier [29], the presence of small white particles noticed on some monofilaments (e.g., [LA]-[H_2_SO_4_-EtOH]-[N]) stems from NaOH contamination from the coagulation bath, which was not removed in the washing process. Figure 2c represents the fibers with the highest tensile strength obtained using lactic acid, citric acid, and acetic acid, respectively (c.f. Figure 3a). All of these fibers were coagulated in a NaOH-EtOH bath and their surface structures were entirely uniform and smooth.

### 3.3. Mechanical Properties

The mechanical properties of the monofilaments from the different fabrication processes were evaluated by tensile testing. All of the measurements were performed at room temperature, after preconditioning the samples for 24 h at 20 °C, and 65% relative humidity. In general, the monofilaments prepared by utilizing the new solvent adipic acid showed significantly higher tensile strength and Young’s modulus, in comparison to the filaments made using other conventional solvents. The exception was utilization of NaOH-EtOH as a coagulation bath without dewatering. Under these conditions the tensile strength properties for fibers produced with lactic acid, acetic acid, and adipic acid were very similar to each other, i.e., 151.7 (±8.7) MPa, 150.1 (±9.8) MPa, and 147.9 (±6.9) MPa, respectively, while a significantly lower tensile strength was obtained when the monofilaments were wet spun from citric acid (110.7 MPa). (Figure 3a).

Moreover, the samples [AD]-[NaOH-EtOH]-[N] and [AD]-[NaOH-EtOH]-[Y] had the highest Young’s modulus of 4.5 GPa and 4.1 GPa, respectively (Figure 3b). This illustrates that the preeminent solvent and coagulation bath, from a strength and modulus perspective, were adipic acid and NaOH-EtOH, respectively. The largest elongation at break (9.5%) was obtained when lactic acid was used as the solvent and NaOH-EtOH was used as coagulation bath (Figure 3c). However, the elongation at break of fibers wet spun with adipic acid was at the same level (8%). Another subtle point is that, although sulfuric acid-derived fibers were less stable in the wet form, they showed promising mechanical strength with the highest tensile strength of 141.7 MPa, which is comparable with the highest value for adipic acid monofilaments.

Tensile strength of all ethanol dewatered monofilaments decreased, regardless of solvent types and coagulation bath composition. This may be explained by the higher internal porosity of the fibers, introduced when dewatered in the EtOH bath. The higher porosity could be due to lower polarity of ethanol compared to water, which reduces the capillary forces acting on chitosan chains during the drying. A similar phenomenon has been reported for nanocellulose membranes subjected to ethanol dewatering [30,31]. Furthermore, elongation at break of most fibers was reduced after experiencing a dewatering bath (c.f. Table 3, Table 4, Table 5 and Table 6). Ethanol-dried monofilaments had larger diameters compared to those from the water bath, as the immediate removal of water with ethanol allowed a more porous fiber and a larger diameter, mostly due to the entrapment of the drying agent in the dehydrant molecules [17]. However, when the fiber was dried at room temperature (without dewatering using EtOH), a more compact fiber was formed. This is probably due to the longer time required for the drying at room temperature, which may lead to the formation of stronger interaction between the chitosan chains.

#### 3.3.1. Monofilaments Made Using Adipic Acid

When adipic acid was used as the solvent, monofilaments with diameters between 165 and 286 micrometers were obtained using different coagulation baths and drying conditions. Dehydration using ethanol increased the diameters significantly. The tensile strength of the fibers using AD was between 74.2 and 147.9 MPa, and the fibers made without dehydration illustrated 18% (for NaOH), 46% (for NaOH-EtOH), and 91% (for H_2_SO_4_-EtOH) higher tensile strength compared to those made with dehydration. Considering all the evaluated acids, when the coagulation bath was either NaOH or H_2_SO_4_-EtOH, by far the highest tensile strength and Young’s modulus were achieved using adipic acid as a solvent. For adipic acid samples, both NaOH and NaOH-EtOH baths generally led to obtaining monofilaments with a better tensile strength compared to fibers made using other acids (LA, CI, and AC). Therefore, adipic acid worked as a good solvent and the resulting fibers had, in many cases, even better properties than those obtained by wet spinning from conventional acids. From the mechanical strength perspective, the best fibers were obtained from [AD]-[NaOH-EtOH]-[N] (Table 3). In addition, although monofilaments coagulated in H_2_SO_4_-EtOH were less stable, the ethanol-dried form of such fibers exhibited a very high tensile strength compared to the other adipic acid fibers.

It was reported that in porous chitosan membranes, adipic acid contributed to more flexible membranes due to the longer carbon backbone of adipic acid compared to other tested acids (acetic acid, oxalic acid, succinic acid, and malic acid) [32]. Furthermore, chitosan and adipic acid showed stronger interactions in the solution and in the fabricated membranes, resulting in higher mechanical strengths. Thus, adipic acid acted as a good solvent for chitosan, but it also brought additional advantages such as acting as a crosslinking reagent for chitosan [32]. Moreover, using adipic acid for production of chitosan films prevented loss of strength and modulus compared to chitosan films made with acetic acid [21]. This is explained by hydrogen bonds and ionic interactions between chitosan and adipic acid, which can set the stage for the physical crosslinking of chitosan by adipic acid. This type of crosslinking reduces the ability of chitosan chains for slippage, leading to a significant increase in the Young’s modulus. The range of Young’s modulus and elongation at break of adipic acid monofilaments were 1.23–4.45 GPa and 1.8% to 8.1%, respectively, depending on the fabrication conditions

#### 3.3.2. Monofilaments Made Using Lactic Acid

The mechanical properties of the fibers produced by using lactic acid as a solvent, generally had somewhat inferior mechanical properties compared to those produced by wet spinning from adipic acid. Within the series of fibers wet spun from lactic acid, the NaOH-EtOH coagulation bath without dewatering (Table 4) resulted in the highest tensile strength by far, which was also comparable with fibers made using adipic acid. Similar to adipic acid fibers, dehydration reduced the tensile strength of the fibers. Moreover, [LA]-[NaOH-EtOH]-[N] showed the highest flexibility in terms of elongation at break, which was 9.5% and marginally higher than the highest elongation at break for adipic acid fibers (8%). Young’s modulus of lactic acid monofilaments was between 1.41 GPa and 3.62 GPa.

#### 3.3.3. Monofilaments Made Using Citric Acid

Even considering citric acid monofilaments, the coagulation bath NaOH-EtOH resulted in the highest strength. (Table 5). For the fibers produced with citric acid, [CI]-[NaOH-EtOH]-[N] and [CI]-[H_2_SO_4_-EtOH]-[N] showed both the highest tensile strength and elongation at break. However, the mechanical performance of these fibers was not at the same level with adipic acid and lactic acid derived samples. More than the stoichiometric amount (0.239 mol/L) of citric acid was required to achieve a proper chitosan dissolution. As a consequence, chitosan solution with citric acid had a lower pH compared to other chitosan solutions (c.f. Table 1). Lower pH might have affected the properties, but further investigations are needed. Additionally, fiber diameters were generally larger compared to the fibers wet spun from the other acids. The range of Young’s modulus and elongation at break of citric acid fibers were 0.58 to 3.41 GPa and 2.1% to 4.7%, respectively.

#### 3.3.4. Monofilaments Made Using Acetic Acid

The fibers wet spun from acetic acid generally had inferior mechanical properties compared to the fibers wet spun from adipic acid. Once again, the monofilaments produced with NaOH-EtOH as coagulation bath illustrated the highest tensile strength (Table 6). Consequently, the only monofilament with high tensile strength comparable to monofilament produced by utilizing adipic acid as a solvent was [AC]-[NaOH-EtOH]-[N]. Furthermore, [AC]-[NaOH-EtOH]-[Y] showed the second highest elongation at break (8.9%) among all monofilaments. Even here, the ethanol-dried fibers had the lowest tensile strength. The range of Young’s modulus and elongation at break of acetic acid monofilaments were 0.88 to 3.74 GPa and 1.3% to 8.9%, respectively.

In this study, the highest tensile strength of the produced adipic acid fibers was 147.9 MPa, which is higher than the strength reported earlier for wet spun chitosan fiber using formic acid (126 MPa [19]), and the wet spun fungal chitosan fiber using lactic acid (72.3 MPa [33]), as solvents. However, the strength was lower than that of wet spun chitosan monofilament using lactic acid (261 MPa) as reported by Costa Da Silva et al. [7]. This difference may be related to the use of methanol in the coagulation bath (70% aqueous solution of 1 M NaOH and 30% methanol). Knaul et al. also reported higher tensile strength for methanol-dried chitosan fibers compared to the fibers obtained by using ethanol as a coagulation bath [17]. Methanol was not used in the current study due to the high toxicity [34]. The highest Young’s modulus obtained here with adipic acid as a solvent was 4.52 GPa, which is lower than some Young’s modulus values for chitosan fibers reported earlier (25.2 GPa [7]). The value is, however, comparable with Young’s modulus of wet spun fungal chitosan fibers produced by Svensson et al. (4.97 GPa) [33]. An elongation at break of 8.1% was achieved in this study with adipic acid as a solvent. This value is in the same range or higher than the elongation at break of wet spun chitosan fibers reported earlier (8.9% [7] and 5.7% [5]). Bigger values of elongation at break (31.5%, 23.1%, and 11.3%) for wet spun chitosan fibers were reported by Han et al. [19], Fan et al. [27], and Tamura et al. [16], respectively. It is expected that the stretching of fibers in different baths and optimizing of the draw ratio [15,35] can contribute to higher tensile strength and Young’s modulus of chitosan fiber wet spun from adipic acid. Dresvyanina et al. [36] reported that tensile strength and Young’s modulus of chitosan monofilaments were around 168 MPa and 6.33 Gpa, respectively, when fibers did not experience stretching. Conversely, for the same condition of spinning (feed rate, shear rate, and precipitation time of chitosan solution), tensile strength and Young’s modulus of chitosan fibers changed to 219 MPa and 7.93 GPa, respectively, when the degree of stretching was 100% [36]. Therefore, the mechanical strength of chitosan monofilaments produced in this study can be improved by optimization of other factors.

### 3.4. Chemical Structure of the Fabricated Monofilaments

**Figure 4:**
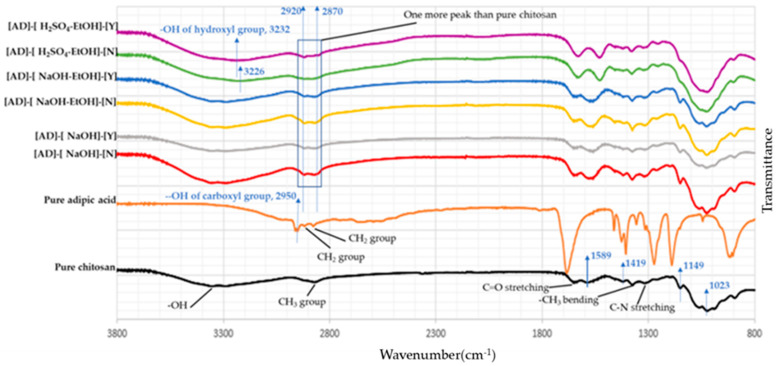
FTIR spectra of adipic acid monofilaments, pure adipic acid and chitosan.

FTIR spectroscopy was used to evaluate the chemical structure of the fabricated monofilaments and the original chitosan powder. Figure 4 shows the vibrational spectra of pure chitosan and the wet spun monofilaments, which were produced using the modified process with adipic acid as a new promising solvent. For pure chitosan, the characteristic absorption bands displayed at 3357 cm^−1^ and 2872 cm^−1^ corresponded to the -OH group and CH_3_ groups, respectively [37]. Bands that appeared at 1647 cm^−1^, 1589 cm^−1^, and 1320 cm^−1^ were related to C=O stretching (amide I), N-H group bending vibration of the primary amine, and C-N stretching (amide III), respectively. Moreover, bands identified at 1419 cm^−1^ and 1374 cm^−1^ were attributed to -CH_2_ bending and -CH_3_ bending absorption, respectively [38]. The bands at 1149 cm^−1^ and 1023 cm^−1^ were related to the stretching of C-O groups [5]. Considering adipic acid samples, most of the absorption bands appeared at the same regions as those of pure chitosan vibrational spectra, except for two differences. Firstly, around 2870–2920 cm^−1^, two absorption bands were identified for all samples prepared with adipic acid as a solvent. However, only one absorption band was seen in this area for pure chitosan sample. Moreover, considering pure adipic acid spectrum, bands which appeared at 2877 cm^−1^ and 2917 cm^−1^ were related to -CH_2_ groups of adipic acid. As a result, the additional band can result from the presence of adipic acid as a crosslinker between chitosan chains [21]. Secondly, the absorption band of -OH group for [AD]-[H_2_SO_4_-EtOH]-[N] at 3226 cm^−1^ and [AD]-[H_2_SO_4_-EtOH]-[Y] at 3232 cm^−1^ became remarkably wider. As previously mentioned, the -OH group of pure chitosan had an absorption band at 3357 cm^−1^. Furthermore, the band as seen at 2950 cm^−1^ of adipic acid spectrum corresponded to the carboxyl group -OH. Therefore, this further supports that adipic acid could act as a crosslinker, contributing to the shift of the hydroxyl absorption band. It should be mentioned that the spectra of fibers made using adipic acid falls in line with the spectra of fibers made using other acids (data not shown).

### 3.5. Thermal Properties of Chitosan Monofilaments

**Figure 5:**
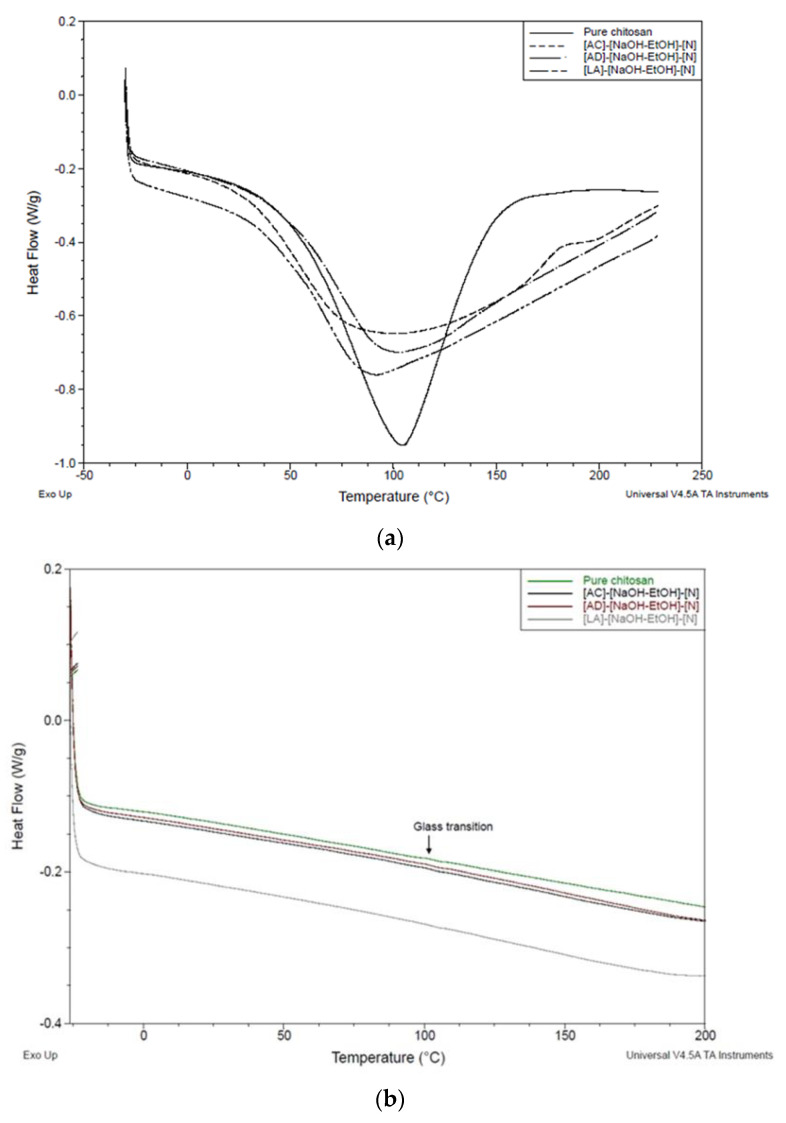
DSC traces of chitosan fibers and pure chitosan: (**a**) first heating run and (**b**) second heating run.

Figure 5 shows the thermograms of pure chitosan and chitosan fibers with the highest tensile strength, which are fibers from adipic, lactic, and acetic acids coagulated in NaOH-EtOH without dewatering. Figure 5a is related to the first heating run. This heating run gives endothermic peaks, which can result from absorbed moisture [39]. For pure chitosan, adipic acid, and acetic acid fibers, peaks were seen at around 105 °C, whereas for lactic acid sample the peak was at about 92 °C. As is represented in Figure 5b, all four peaks disappeared, supporting the idea that water evaporation occurred during the first DSC run. In order to remove the effect of moisture on glass transition temperature (Tg) measurement, the second heating run was considered, showing the small changes in inclination of the baseline at about 105 °C. While this value could possibly attribute to the Tg of chitosan powder and chitosan fiber, it is in contrast to the value of Tg reported earlier [39]. Different treatments using different acids did not lead to a significant change in the Tg of chitosan fibers compared to pure chitosan. It should be noted that the Tg of the common polymers can be found by DSC. However, Tg of chitosan is still a controversial subject. The major reason may stem from the fact that some properties of chitosan (e.g., crystallinity, molecular weight, and deacetylation degree) as a natural polymer can show wide variations due to the source and/or method of extraction, influencing the Tg [40]. To clarify further, Ratto et al. [41] reported that the Tg of chitosan is at 30 °C for water contents between 8 and 30%, while Lazaridou and Biliaderis [42] observed that Tg ranged from −23 to 67 °C. The Tg of chitosan at a higher temperature, 203 °C, was also reported by Sakurai et al. [43]. Therefore, the heterogenicity of the polymers such as chitosan leads to some difficulties in finding the Tg.

## 4. Conclusions

Chitosan monofilaments are usually created by wet spinning using lactic acid, citric acid, or acetic acid as a solvent. Our results reveal that adipic acid, has great potential as a solvent for production of chitosan monofilaments. Monofilaments produced with adipic acid as a solvent generally illustrated higher tensile strength (tensile strength of 147.9 MPa and Young’s modulus of 4.45 GPa) compared to the monofilaments produced with commonly used acid solvents under the same conditions. This is explained by the potential of di-functional adipic acid to form physical crosslinks between chitosan chains according to the additional band seen at around 2870–2920 cm^−1^, which was assigned to the -CH_2_ group of adipic acid. In addition, the properties of the monofilaments were influenced by different coagulation baths. Fibers with higher mechanical strength were obtained when NaOH-EtOH was used as the coagulation bath. Interestingly, a mixture of aqueous H_2_SO_4_ and EtOH could coagulate chitosan solutions, contributing to the fabrication of uniform and smooth monofilaments.

## Figures and Tables

**Table 1:** pH and viscosity of different chitosan solutions.

Solvents’ ID	Type of Solvent	pH	Viscosity (Pa.s)
LA	Lactic acid	4.5	3.0
AD	Adipic acid	4.8	3.3
CI	Citric acid	4.2	2.4
AC	Acetic acid	5.5	3.3

**Table 2:** The different conditions in which fibers were produced.

Samples’ ID	Solvent	Coagulation Bath	Dewatering (EtOH)
[LA]-[NaOH]-[N]	Lactic acid	NaOH 1 M	No
[LA]-[NaOH]-[Y]	Lactic acid	NaOH 1 M	Yes
[LA]-[NaOH-EtOH]-[N]	Lactic acid	(1:1) 10% solution of NaOH and EtOH	No
[LA]-[NaOH-EtOH]-[Y]	Lactic acid	(1:1) 10% solution of NaOH and EtOH	Yes
[LA]-[H_2_SO_4_-EtOH]-[N]	Lactic acid	(1:1) 2% solution of H_2_SO_4_ and EtOH	No
[LA]-[H_2_SO_4_-EtOH]-[Y]	Lactic acid	(1:1) 2% solution of H_2_SO_4_ and EtOH	Yes
[AD]-[NaOH]-[N]	Adipic acid	NaOH 1 M	No
[AD]-[NaOH]-[Y]	Adipic acid	NaOH 1 M	Yes
[AD]-[NaOH-EtOH]-[N]	Adipic acid	(1:1) 10% solution of NaOH and EtOH	No
[AD]-[NaOH-EtOH]-[Y]	Adipic acid	(1:1) 10% solution of NaOH and EtOH	Yes
[AD]-[H_2_SO_4_-EtOH]-[N]	Adipic acid	(1:1) 2% solution of H_2_SO_4_ and EtOH	No
[AD]-[H_2_SO_4_-EtOH]-[Y]	Adipic acid	(1:1) 2% solution of H_2_SO_4_ and EtOH	Yes
[CI]-[NaOH]-[N]	Citric acid	NaOH 1 M	No
[CI]-[NaOH]-[Y]	Citric acid	NaOH 1 M	Yes
[CI]-[NaOH-EtOH]-[N]	Citric acid	(1:1) 10% solution of NaOH and EtOH	No
[CI]-[NaOH-EtOH]-[Y]	Citric acid	(1:1) 10% solution of NaOH and EtOH	Yes
[CI]-[H_2_SO_4_-EtOH]-[N]	Citric acid	(1:1) 2% solution of H2SO_4_ and EtOH	No
[CI]-[H_2_SO_4_-EtOH]-[Y]	Citric acid	(1:1) 2% solution of H2SO_4_ and EtOH	Yes
[AC]-[NaOH]-[N]	Acetic acid	NaOH 1 M	No
[AC]-[NaOH]-[Y]	Acetic acid	NaOH 1 M	Yes
[AC]-[NaOH-EtOH]-[N]	Acetic acid	(1:1) 10% solution of NaOH and EtOH	No
[AC]-[NaOH-EtOH]-[Y]	Acetic acid	(1:1) 10% solution of NaOH and EtOH	Yes
[AC]-[H_2_SO_4_-EtOH]-[N]	Acetic acid	(1:1) 2% solution of H_2_SO_4_ and EtOH	No
[AC]-[H_2_SO_4_-EtOH]-[Y]	Acetic acid	(1:1) 2% solution of H_2_SO_4_ and EtOH	Yes

**Table 3:** Mechanical properties of wet spun monofilaments using adipic acid as the solvent.

Sample	Diameter (mm)	Young’s Modulus (GPa)	Tensile Strength (MPa)	Elongation at Break (%)
[AD]-[NaOH]-[N]	0.165 (±0.02)	4.11 (±0.7)	137.5 (±9.1)	6.5 (±2.1)
[AD]-[NaOH]-[Y]	0.221 (±0.03)	1.23 (± 0.5)	116.8 (± 6.3)	5.2 (± 1.9)
[AD]-[NaOH-EtOH]-[N]	0.183 (±0.01)	4.45 (±0.7)	147.9 (±6.9)	8.0 (±1.1)
[AD]-[NaOH-EtOH]-[Y]	0.231 (±0.01)	4.15 (±0.9)	101.4 (±6.7)	8.1 (±2.3)
[AD]-[H_2_SO_4_-EtOH]-[N]	0.197 (±0.03)	3.17 (±0.3)	141.7 (±4.9)	1.9 (±0.8)
[AD]-[H_2_SO_4_-EtOH]-[Y]	0.286 (±0.02)	2.13 (±0.5)	74.2 (±1.3)	1.8 (±0.9)

**Table 4:** Mechanical properties of wet spun monofilaments using lactic acid as the solvent.

Sample	Diameter (mm)	Young’s Modulus (Gpa)	Tensile STRENGTH (Mpa)	Elongation at Break (%)
[LA]-[NaOH]-[N]	0.193 (±0.01)	3.62 (±0.2)	89.5 (±2.6)	3.3 (±1.0)
[LA]-[NaOH]-[Y]	0.235 (±0.02)	2.49 (±0.2)	58.8 (±4.9)	2.9 (±0.3)
[LA]-[NaOH-EtOH]-[N]	0.187 (±0.03)	1.41 (±0.5)	151.7 (±8.7)	9.5 (±1.2)
[LA]-[NaOH-EtOH]-[Y]	0.226 (±0.01)	3.07 (±1.0)	76.6 (±4.6)	7.0 (±2.6)
[LA]-[H_2_SO_4_-EtOH]-[N]	0.217 (±0.02)	3.24 (±0.2)	84.7 (±2.5)	3.6 (±0.6)
[LA]-[H_2_SO_4_-EtOH]-[Y]	0.269 (±0.01)	1.22 (±0.5)	58.7 (±4.1)	3.3 (±0.7)

**Table 5:** Mechanical properties of wet spun monofilaments using citric acid as the solvent.

Sample	Diameter (mm)	Young’s Modulus (GPa)	Tensile STRENGTH (MPa)	Elongation at Break (%)
[CI]-[NaOH]-[N]	0.186 (±0.01)	2.38 (±0.31)	86.2 (±4.1)	2.9 (±0.4)
[CI]-[NaOH]-[Y]	0.218 (±0.02)	1.75 (±0.91)	50.4 (±4.2)	2.7 (±0.9)
[CI]-[NaOH-EtOH]-[N]	0.264 (±0.01)	3.41 (±1.06)	110.7 (±2.4)	4.7 (±1.0)
[CI]-[NaOH-EtOH]-[Y]	0.275 (±0.03)	0.58 (±0.24)	54.4 (±5.3)	5.0 (±0.6)
[CI]-[H_2_SO_4_-EtOH]-[N]	0.232 (±0.02)	1.75 (±0.32)	106.3 (±7.4)	4.5 (±1.1)
[CI]-[H_2_SO_4_-EtOH]-[Y]	0.293 (±0.01)	0.83 (±0.22)	54.3 (±4.2)	2.1 (±0.8)

**Table 6:** Mechanical properties of wet spun monofilaments using acetic acid as the solvent.

Sample	Diameter (mm)	Young’s Modulus (GPa)	Tensile STRENGTH (MPa)	Elongation at Break (%)
[AC]-[NaOH]-[N]	0.19 (±0.02)	3.74 (±0.52)	92.1 (±4.5)	7.3 (±0.9)
[AC]-[NaOH]-[Y]	0.241 (±0.01)	1.09 (±0.43)	33.2 (±1.1)	1.3 (±0.4)
[AC]-[NaOH-EtOH]-[N]	0.250 (±0.02)	2.37 (±0.83)	150.1 (±9.8)	5.1 (±1.2)
[AC]-[NaOH-EtOH]-[Y]	0.185 (±0.03)	3.68 (±0.82)	97.1 (±8.9)	8.9 (±0.9)
[AC]-[H_2_SO_4_-EtOH]-[N]	0.203 (±0.03)	1.71 (±0.16)	79.6 (±5.6)	4.1 (±0.4)
[AC]-[H_2_SO_4_-EtOH]-[Y]	0.279 (±0.02)	0.88 (±0.19)	70.8 (±3.0)	3.2 (±0.6)

## Data Availability

Not applicable.
